# The Impact of Polyamine Precursors, Polyamines, and Steroid Hormones on Temporal Messenger RNA Abundance in Bovine Satellite Cells Induced to Differentiate

**DOI:** 10.3390/ani11030764

**Published:** 2021-03-10

**Authors:** Caleb C. Reichhardt, Lillian L. Okamoto, Laura A. Motsinger, Brian P. Griffin, Gordon K. Murdoch, Kara J. Thornton

**Affiliations:** 1 Department of Animal, Dairy and Veterinary Science, Utah State University, 4815 Old Main Hill, Logan, UT 84322, USA; ccreichhardt@gmail.com (C.C.R.); lelehua521@gmail.com (L.L.O.); laura_smith95@live.com (L.A.M.); brian.griffin0710@gmail.com (B.P.G.); 2 Department of Animal, Veterinary and Food Sciences, University of Idaho, Logan, ID 83844, USA; gordon.murdoch@wsu.edu; 3 Department of Animal Sciences, Washington State University, Pullman, WA 99163, USA

**Keywords:** anabolic implants, bovine satellite cells, beef, polyamines, skeletal muscle

## Abstract

**Simple Summary:**

In the U.S., approximately 90% of all cattle on feed receive an anabolic implant at some point during production. Despite the widespread use, how they operate to increase growth of cattle remains unknown. Polyamines are amino acid derivatives, which are potent growth stimulants, produced through the polyamine biosynthetic pathway. Emerging research suggests that the hormones in anabolic implants interact with the polyamine biosynthetic pathway. The purpose of this research was to investigate the effects of steroidal hormones, polyamine precursors, and polyamines on mRNA abundance of bovine satellite cells, muscle precursor cells. The results from this study suggest that polyamine precursors and polyamines alter transcription factors involved in induction of differentiation of bovine satellite cells and the polyamine biosynthetic pathway, while the hormones in anabolic implants alter genes involved in the polyamine biosynthetic pathway. These results mean that polyamines may impact differentiation of bovine satellite cells, ultimately affecting growth of cattle.

**Abstract:**

Emerging research suggests that hormones found in anabolic implants interact with polyamine biosynthesis. The objective of this study was to determine the effects of steroidal hormones, polyamines and polyamine precursors on bovine satellite cell (BSC) differentiation and polyamine biosynthesis temporally. Primary BSCs were induced to differentiate in 3% horse serum (CON) and treated with 10 nM trenbolone acetate (TBA), 10 nM estradiol (E2), 10 nM TBA and 10 nM E2, 10 mM methionine, 8 mM ornithine, 2 mM putrescine, 1.5 mM spermidine, or 0.5 mM spermine. Total mRNA was isolated 0, 2, 4, 8, 12, 24, and 48 h post-treatment. Abundance of mRNA for genes associated with induction of BSC differentiation: paired box transcription factor 7, myogenic factor 5, and myogenic differentiation factor 1 and genes in the polyamine biosynthesis pathway: ornithine decarboxylase and S-adenosylmethionine—were analyzed. Overall, steroidal hormones did not impact (*p* > 0.05) mRNA abundance of genes involved in BSC differentiation, but did alter (*p* = 0.04) abundance of genes involved in polyamine biosynthesis. Polyamine precursors influenced (*p* < 0.05) mRNA of genes involved in BSC differentiation. These results indicate that polyamine precursors and polyamines impact BSC differentiation and abundance of mRNA involved in polyamine biosynthesis, while steroidal hormones altered the mRNA involved in polyamine biosynthesis.

## 1. Introduction 

For the livestock industry, skeletal muscle growth is extremely important as, through a series of changes, it becomes meat [1,2]. There is a positive correlation between environmental sustainability and improved productivity [3]. Therefore, understanding mechanisms that improve skeletal muscle growth is necessary to produce cattle that are more environmentally sustainable, while decreasing costs to producers. Mammalian muscle fiber number is primarily fixed at birth, making hypertrophy of existing fibers the primary mechanism for postnatal growth [4,5,6]. However, hypertrophy eventually requires additional nuclei from satellite cells for muscle growth [4,5,6,7]. Satellite cells are muscle precursor cells that proliferate, then differentiate and fuse into myotubes or with existing muscle fibers to support hypertrophy [5]. Differentiation and phenotypic maturation are necessary for satellite cells to fuse [8]. Markers of differentiation in skeletal muscle include increased expression of myogenic differentiation factor 1 *(MyoD*), myogenic factor 5 (*Myf5*), and myogenin, and decreased expression of paired box transcription factor 7 (*Pax7*) [6,9,10].

Anabolic implants are typically composed of estradiol (E2) and/or other compounds such as trenbolone acetate (TBA) and are used to increase the overall efficiency of beef cattle production [7,11,12]. As such, approximately 90% of cattle on feed in the U.S. receive an anabolic implant some point during production [13]. While proven effective, the physiological mechanisms by which TBA and E2 function to increase growth in cattle are not fully understood [14,15,16,17,18,19,20,21]. Treatment of bovine satellite cells (BSCs) with TBA or E2 results in increased proliferation and protein synthesis rates [14,15,16,17,18,19,20,21]. Previous research investigating the effects of steroidal hormones on BSCs has established that both E2 and TBA increase fusion rates at both 48 and 72 h post-treatment [22]. However, knowledge gaps regarding anabolic implants and growth of skeletal muscle still remain. 

Emerging research has demonstrated that TBA and E2 may interact with the polyamine biosynthetic pathway [23,24,25]. Polyamines, including putrescine (PUT), spermidine (SPD), and spermine (SPE), are important for normal cell growth and differentiation [26,27,28,29,30,31]. Polyamines are orally active, and found in relatively high concentrations in common feedstuffs, such as silage [29]. Research shows that when BSCs are treated with polyamines or polyamine precursors, proliferation rates increase [25]. The major substrates used in the polyamine biosynthesis pathway to produce PUT, SPD, and SPE are methionine (MET), ornithine (ORN), and arginine [32]. The production of PUT from ORN, catalyzed by ornithine decarboxylase (ODC), is the first rate-limiting step in the polyamine biosynthesis pathway [33]. The second rate-limiting step is the production of decarboxylated-S-adenosylmethionine, catalyzed by S-adenosylmethionine decarboxylase (AMD1) [33]. In proliferating BSCs, TBA has been shown to interact with the polyamine biosynthesis pathway [25].

The interactions of steroidal hormones, polyamine precursors, polyamines, and the polyamine biosynthetic pathway have not been well characterized in differentiating BSCs. Therefore, the objective of this research was to investigate whether steroidal hormones (TBA and E2), polyamines (PUT, SPD and SPE), and polyamine precursors (MET and ORN) influence mRNA abundance of BSCs induced to differentiate and the polyamine biosynthesis pathway temporally. 

## 2. Materials and Methods 

### 2.1. Bovine Satellite Cell Isolation

Bovine satellite cells were isolated from three different steers raised and harvested following procedures approved by the Institutional Animal Care and Use Committee (IACUC Protocol # 10216) at Utah State University. These steers were approximately one year of age and weighed approximately 315 kg at harvest and had not previously received any anabolic implants or other growth promotants. Animals were euthanized by captive bolt, followed by exsanguination. Bovine satellite cell isolation was performed as previously described [15,16,18,34,35]. In brief, using sterile techniques, one kg of the *semimembranosus* was collected and transported approximately 12 km to the laboratory. Approximately 45 m elapsed from exsanguination to the start of BSC isolation. The following was conducted using aseptic techniques in a tissue culture hood. Adipose and connective tissue were removed, and the muscle was passed through a sterile meat grinder. The ground muscle was then incubated with a 0.1% pronase in Earl’s Balanced Salt Solution for 1 h at 37 °C and mixed every 10 min. The mixture was then centrifuged at 1500× *g* for 4 min at 4 °C and the resultant pellets were resuspended in phosphate-buffered saline solution (PBS: 140 mM NaCl, 1.0 mM KH_2_PO_4,_ 3.0 mM KCl, and 8.0 MM Na_2_HPO_4_) and centrifuged again at 500× *g* for 10 min. The recovered supernatant was centrifuged at 1500× *g* for 10 min to pellet the mononucleated cells. The PBS wash and centrifugation were repeated two more times. The monocucleated cell preparation was suspended in 4 °C Dulbecco’s Modified Eagle Medium (DMEM), containing 10% fetal bovine serum (FBS), and 10% dimethylsulfoxide (DMSO), and then frozen at −80 °C. The cells were stored in liquid nitrogen until subsequent use. Clonal analysis of satellite cell cultures established from these preparations showed that between 80% and 90% of the cells in these preparations were myogenic. Phenol red was not present in any of the culture media used in this study, as phenol red is known to interact with the androgen and estrogen receptors. 

### 2.2. Bovine Satellite Cell Culture

Bovine satellite cells were plated as previously described [16]. In brief, BSC cultures were plated in 4 cm^2^ wells that were precoated with reduced growth factor basement membrane Matrigel (Corning, Tewksbury, MA, USA) diluted 1:50 (*v*/*v*). Cultures were plated at a density of 2 g/cm^2^, which produced cultures that were approximately 70% confluent after 72 h in culture. Cells were plated in Dulbecco’s Modified Eagle Medium (DMEM) containing 10% FBS and incubated at 37 °C with 5% CO_2_ in a water saturated environment [16]. At 72 h, cultures were rinsed twice with DMEM and fresh media with 10% FBS was added to cultures. Cultured cells from each of the three animals used in the study were completed in duplicate, providing a total of six samples per time point per treatment.

### 2.3. Induction of Differentiation and Treatment of BSC Cultures 

Bovine satellite cell cultures were grown until a confluency of approximately 80% was reached, at which point the cells were induced to differentiate in DMEM containing 3% horse serum and 1.5% bovine serum albumin-linoleic acid (BSA-LA) following previous described methods [17], and containing the treatments. Treatments were added to differentiation media and consisted of a control (CON), steroidal hormones (10 nM TBA, 10 nM E2 or 10 nM TBA and 10 nM E2), polyamines (2 mM PUT, 0.5 mM SPE, or 1.5 mM SPD) or polyamine precursors (8 mM ORN or 10 mM MET). The treatment concentrations were chosen based on previous research examining polyamine levels in bovine lymphocytes [27], as no research has been completed in bovine skeletal muscle cells, and experiments previously conducted by our lab group demonstrating that these concentrations increase BSC proliferation [25].

### 2.4. RNA Isolation, Quantification, and cDNA Synthesis

Total RNA was extracted from BSC cultures using the Absolutely RNA Microprep Kit (Agilent Technologies, Cedar Creek, TX, USA) as per the manufacturer’s protocol. Cells were lysed at 0, 2, 4, 8, 12, 24, and 48 h post-treatment. In brief, lysis buffer was added directly to the culture dish and a cell scraper was used to further lyse the cells. The cell lysate was then vortexed and an equal volume of 70% ethanol was added. The mixture was then centrifuged and filtered. A series of wash buffers were added and then the RNA was eluted and stored at −80 °C. Isolated RNA was quantified using a BioTek all-in-one microplate reader using Gen5 2.0 software (BioTek Instruments, Winooski, VT, USA), and quality was determined using the 260/280 ratio. Samples with a ratio greater than 2.0 were deemed high enough quality for cDNA synthesis. All RNA samples were treated with deoxyribonuclease (Ambion, Foster City, CA, USA) before beginning cDNA synthesis using a high-capacity cDNA reverse transcription kit (Applied Biosystems, Foster City, CA, USA) following the manufacturer’s protocol. 

### 2.5. Quantitative Real-Time PCR

Real-time PCR quantification of mRNA was assessed using the Taqman MGB primer/probe system. Primer express 3.0 software (Applied Biosystems, Foster City, CA, USA) was used to design the primers and probes for all genes. A list of primers can be found in Table 1. An ABI 7500 real-time PCR system (Applied Biosystems, Foster City, CA, USA) was used to detect relative mRNA abundance of Ribosomal 18S (*18S)*, *ODC, AMD1, PAX7, MYF5*, or *MYOD.* All samples were analyzed in duplicate. If the Ct of the duplicates differed by more than 0.5, the sample was reanalyzed. Ribosomal 18S was used as the housekeeping gene using the per sample ∆C_t_ method [36,37,38]. Ribosomal 18S has been found to be within the range of acceptance for stability in bovine muscle tissue [38] and is commonly used as a housekeeping gene in experiments analyzing cultured skeletal muscle cells [39,40].

### 2.6. Statistical Analysis 

**Figure 1:**
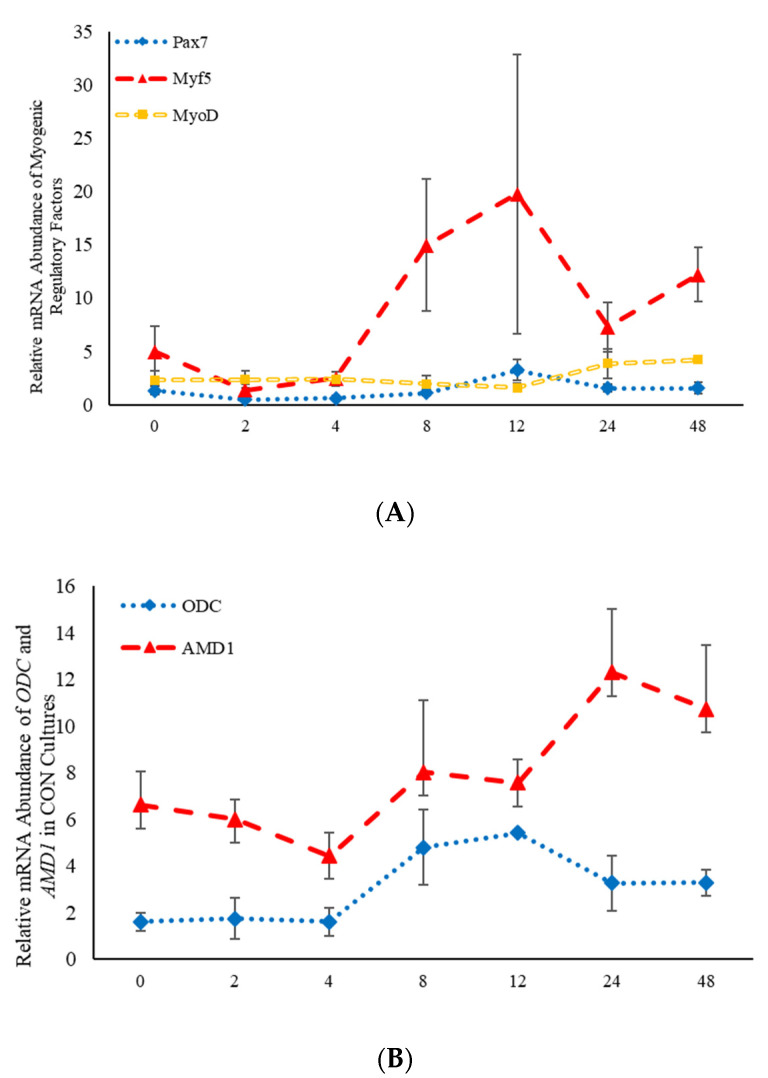
Relative mRNA abundance of the myogenic regulatory factors (**A**): paired box transcription factor 7 (*Pax7*)*,* myogenic factor 5 *(Myf5*)*,* and myogenic differentiation factor 1 *(MyoD*) and relative mRNA abundance of the two rate limiting steps involved in the polyamine biosynthesis pathway (**B**): S-adenosylmethionine decarboxylase (AMD1) and ornithine decarboxylase (ODC) from control-treated primary bovine satellite cells cultures over time. Abundance was measured 0, 2, 4, 8, 12, 24 and 48 h after treatment, as described in the Materials and Methods.

Statistical analysis was performed using the MIXED procedure of SAS (version 9.4; SAS Inst. Inc., Cary, NC, USA). All data are presented as the least square mean ± standard error of the mean (SEM). Data from multiple assays performed on cells isolated from different animals were combined. Preliminary analyses indicated that there were no effects (*p* > 0.05) observed for either the assay number or the different animals, and as such, these two factors were included as random variables in the model. Three separate analyses were completed to determine the effects of steroid hormones, polyamines, and polyamine precursors over time. As samples were analyzed over time, a repeated-measures analysis was performed to assess the fixed effects of treatment (steroidal hormone, polyamine or polyamine precursor), time, and treatment*time. When treatment differences were found to be significant (*p* < 0.05), contrasts were constructed to determine whether each of the nine different treatments were different from the control cultures. Relative mRNA abundance of the CON cultures over time can be found in Figure 1. Tukey–Kramer adjustments were made in both the repeated-measures analysis, as well as in the contrast statements to control for multiple comparisons. This analysis was performed for data obtained from quantitative real-time PCR. Gene expression analysis using TAQman quantitative real-time PCR was performed by analyzing the relative expression of each sample calculated as 2-relative threshold cycle (ΔCt).

## 3. Results

### 3.1. The Effects of Steroidal Hormones on Abundance of mRNA Involved in BSC Differentiation

**Figure 2:**
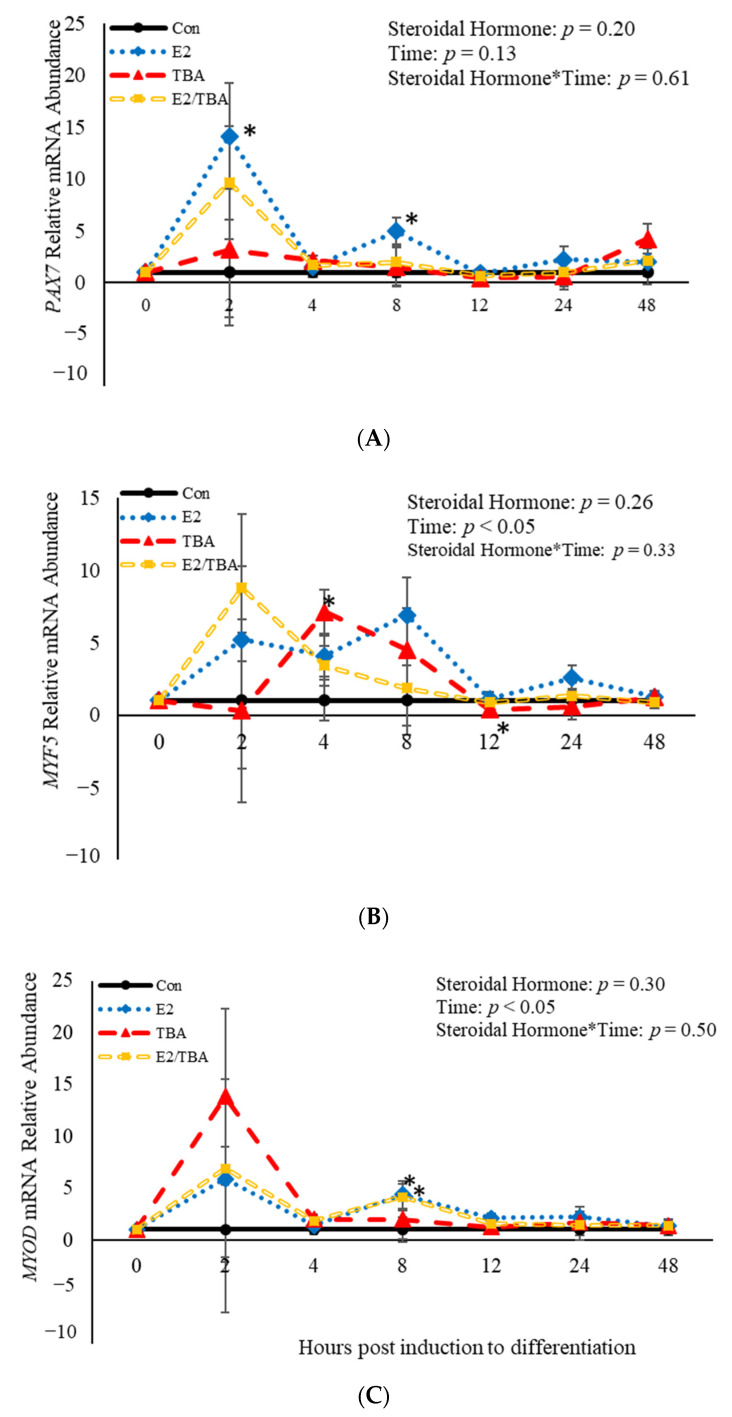
Relative mRNA abundance of paired box transcription factor 7 (*PAX7*; (**A**)*,* myogenic factor 5 *(MYF5;* (**B**), and myogenic differentiation factor 1 *(MYOD*; (**C**) from primary bovine satellite cells cultures following treatment with 10 nM trenbolone acetate (TBA), 10 nM estradiol (E2), or 10 nM E2 and 10 nM TBA (E2/TBA). Cultures were grown to 80% confluency and treated with DMEM/3% horse serum and 10 nM TBA, 10 nM E2, or 10 nM E2/TBA. Abundance was measured 0, 2, 4, 8, 12, 24 and 48 h after treatment, as described in the Materials and Methods. The main effects of steroidal hormone, time and steroidal hormone*time in mRNA abundance were analyzed as repeated-measures. Data represent relative mRNA abundance, normalized against 18S abundance and are presented as LS mean ± SEM from six separate replicates utilizing BSCs isolated BSCs isolated from at least three different animals. Differences (*p* < 0.05) between steroidal hormones found in anabolic implants and the control cultures are indicated by a * at that particular time point.

No effects (*p* > 0.05) of steroidal hormone or steroidal hormone*time were observed relative to abundance of *PAX7, MYF5,* or *MYOD* mRNA at 2, 4, 8, 12, 24, or 48 h post-treatment (Figure 2). However, treatment with E2 increased abundance of *PAX7* mRNA at both 2 (*p* = 0.02) and 8 h (*p =* 0.04) when compared to control cultures (Figure 2A). Trenbolone acetate increased (*p* = 0.001) mRNA abundance of *MYF5* 4 h post-induction to differentiate and decreased (*p* = 0.04) *MYF5* abundance at 12 h post-induction to differentiate compared to control cultures (Figure 2B). Additionally, both E2 (*p* = 0.02) and ETBA (*p =* 0.03) increased abundance of *MYOD* mRNA 8 h post-induction to differentiate (Figure 1A). Abundance of *MYF5* and *MYOD* mRNA was affected (*p* < 0.05) by time (Figure 2). Abundance of *MYF5* increased (*p* = 0.02) for the first 8 h of induction to differentiate and then decreased after that time (Figure 2B). A similar pattern was observed in abundance of *MYOD* mRNA such that it increased initially, at 2 h post-induction to differentiate, and decreased after that time (Figure 2C). Myogenin was not expressed by any of the cultures at any time point assessed. These data demonstrate that the steroidal hormones found in anabolic implants increase abundance of *MYF5* at 4 and 8 h, respectively, post-induction to differentiate. Furthermore, mRNA abundance of *MYF5* and *MYOD* is altered in BSCs temporally. 

### 3.2. The Effects of Steroidal Hormones on Abundance of mRNA Involved in the Polyamine Biosynthesis Pathway

**Figure 3:**
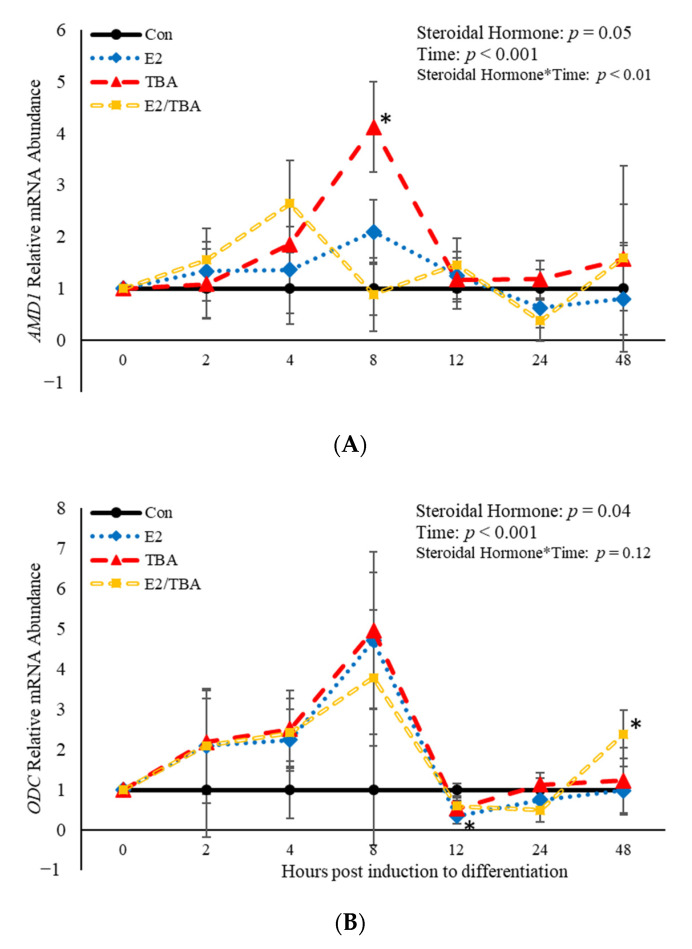
Relative mRNA abundance of *S-adenosylmethionine decarboxylase* (*AMD1;* (**A**) and *ornithine decarboxylase (ODC;* (**B**) from primary bovine satellite cells cultures following treatment with 10 nM trenbolone acetate (TBA), 10 nM estradiol (E2), or 10 nM E2 and 10 nM TBA (E2/TBA). Cultures were grown to 80% confluency and treated with DMEM/3% horse serum and 10 nM TBA, 10 nM E2, or 10 nM E2/TBA. Abundance was measured 0, 2, 4, 8, 12, 24 and 48 h after treatment, as described in the Materials and Methods. The main effects of steroidal hormone, time and steroidal hormone *time in mRNA abundance were analyzed as repeated-measures. Data represent relative mRNA abundance normalized against 18S abundance and are presented as LS mean ± SEM from six separate replicates utilizing BSCs isolated BSCs isolated from at least three different animals. Differences (*p* < 0.05) between steroidal hormones found in anabolic implants and the control cultures are indicated by a * at that particular time point.

The mRNA abundance of genes involved in the polyamine biosynthesis pathway (*ODC* and *AMD1*) was affected (*p* < 0.05) by treatment with the hormones found in anabolic implants (Figure 3). Additionally, a steroidal hormone*time interaction (*p* = 0.003) was observed for *AMD1* (Figure 3A). Eight hours post-induction to differentiate, *AMD1* abundance was increased (*p* = 0.006) when BSCs were treated with TBA (Figure 3A). Estradiol decreased (*p* = 0.02) abundance of *ODC* 12 h post-induction to differentiate (Figure 3B) compared to control cultures. There was also an effect (*p* < 0.05) of time on abundance of both *AMD1* and *ODC* (Figure 3). Abundance of *AMD1* increased (*p <* 0.05) at 4 and 8 h post-induction to differentiate, then decreased after that time. Similarly, abundance of *ODC* increased from 2 to 8 h post-induction to differentiate, then decreased after that time point. These data demonstrate that the steroidal hormones found in anabolic implants impact abundance of the two main rate limiting enzymes in the polyamine biosynthesis pathway. Furthermore, abundance of these enzymes changes over time following induction of differentiation.

### 3.3. The Effects of Polyamine Precursors on Abundance of mRNA Related to Satellite Cell Differentiation

**Figure 4:**
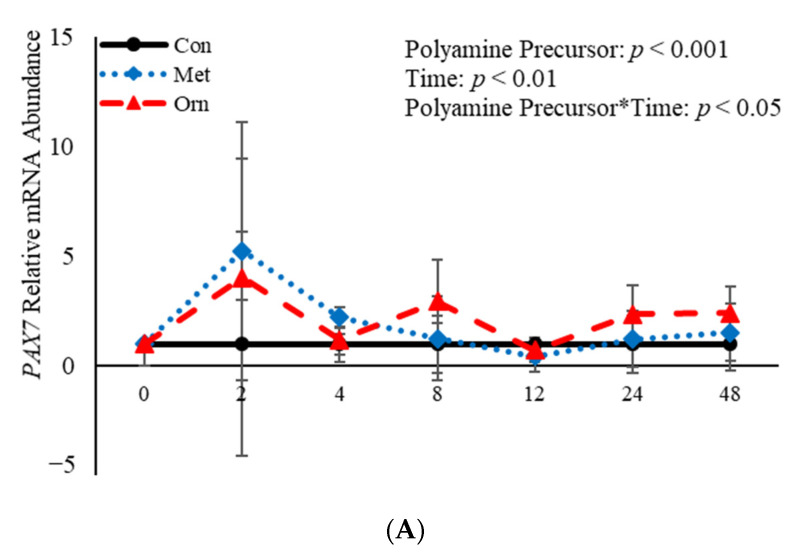

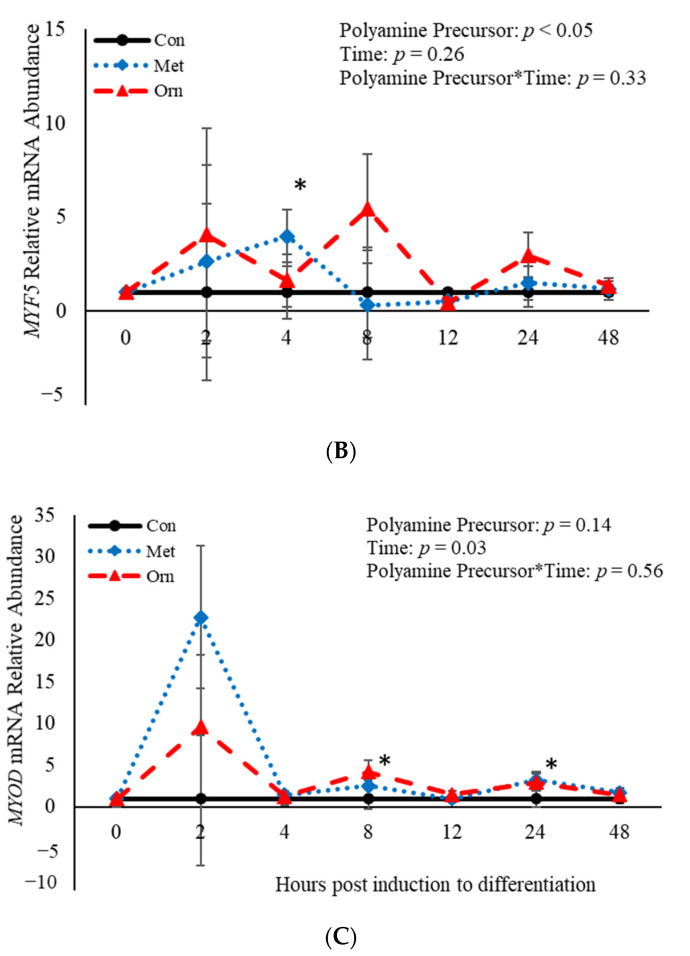
Relative mRNA abundance of paired box transcription factor 7 (*PAX7*; (**A**), myogenic factor 5 *(MYF5;* (**B**), and myogenic differentiation factor 1 *(MYOD*; (**C**) from primary bovine satellite cells cultures following treatment with 10 mM methionine (MET) or 8 mM ornithine (ORN). Cultures were grown to 80% confluency and treated with DMEM/3% horse serum and 10 mM MET or 8 mM ORN. Abundance was measured 0, 2, 4, 8, 12, 24 and 48 h after treatment, as described in the Materials and Methods. The main effects of polyamine precursor, time and polyamine precursor*time in mRNA abundance were analyzed as repeated-measures. Data represent relative mRNA abundance normalized against 18S abundance and are presented as LS mean ± SEM from six separate replicates utilizing BSCs isolated BSCs isolated from at least three different animals. Differences (*p* < 0.05) between polyamine precursors and the control cultures are indicated by a * at that particular time point.

No effect (*p* > 0.05) of the interaction between polyamine precursor*time was observed in mRNA abundance of *MYF5* or *MYOD*, however there was a polyamine precursor*treatment effect (*p* = 0.04) observed for *PAX7* mRNA (Figure 4). Relative mRNA abundance of *PAX7* was affected (*p* < 0.001) by treatment when a polyamine precursor when analyzed as a repeated measure. However, neither polyamine precursor affected (*p* > 0.05) abundance of *PAX7* compared to the control cultures at any of the analyzed time points (Figure 4A). Additionally, mRNA of *MYF5* was affected (*p* = 0.05) by treatment with polyamine precursors (Figure 4B). Compared to control cultures, MET increased (*p* = 0.05) abundance of *MYF5* 4 h post-induction to differentiate (Figure 4B). No effect (*p* > 0.05) of treatment with polyamine precursor was observed in abundance of *MYOD*. However, MET increased (*p* = 0.008) *MYOD* abundance 24 h post-induction to differentiate compared to control cultures (Figure 4C). Additionally, ORN increased mRNA abundance of *MYOD* 8 h (*p* = 0.05) and 24 h (*p* = 0.007) post-induction to differentiate compared to control cultures (Figure 4C). Myogenin was not expressed by any of the cultures at any time point assessed. Abundance of both *PAX7* and *MYOD* was affected (*p* < 0.05) by time such that expression of each gene was increased (*p* < 0.05) 2 h post-induction to differentiate and then decreased after that time point. The data presented here demonstrate that polyamine precursors generally increase abundance of genes involved in BSC differentiation.

### 3.4. The Effects of Polyamine Precursors on Abundance of mRNA Involved in the Polyamine Biosynthesis Pathway

**Figure 5:**
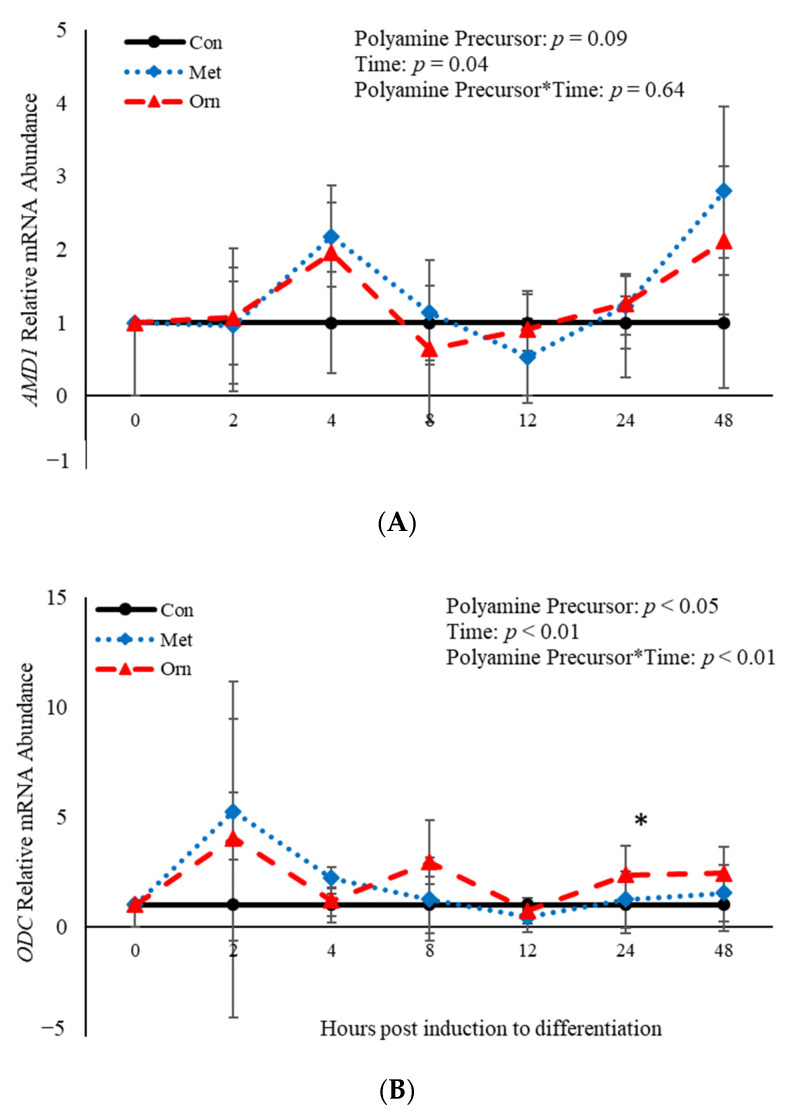
Relative mRNA abundance of S-adenosylmethionine decarboxylase (*AMD1;* (**A**) and ornithine decarboxylase *(ODC;* (**B**) from primary bovine satellite cells cultures following treatment with 10 mM methionine (MET) or 8 mM ornithine (ORN). Cultures were grown to 80% confluency and treated with DMEM/3% horse serum and 10 mM MET or 8 mM ORN. Abundance was measured 0, 2, 4, 8, 12, 24 and 48 h after treatment, as described in the Materials and Methods. The main effects of polyamine precursor, time and polyamine precursor*time in mRNA abundance were analyzed as repeated-measures. Data represent relative mRNA abundance normalized against 18S abundance and are presented as LS mean ± SEM from six separate replicates utilizing BSCs isolated BSCs isolated from at least three different animals. Differences (*p* < 0.05) between polyamine precursors and the control cultures are indicated by a * at that particular time point.

No effect of polyamine precursor (*p* > 0.05) or polyamine precursor*time interaction (*p* > 0.05) was observed relative to *AMD1* mRNA abundance (Figure 5A). However, an effect of polyamine precursor (*p* = 0.03), time (*p* < 0.001) and their interaction (*p =* 0.008) was observed on abundance of *ODC* mRNA (Figure 5B). Twenty-four h post-induction to differentiate, ORN increased (*p* = 0.04) abundance of *ODC* when compared to control cultures (Figure 5B). Additionally, abundance of *AMD1* and *ODC* were both affected (*p* < 0.05) by time (Figure 5). Abundance of *AMD1* increased 4 h after induction of differentiation and then decreased after that time, whereas abundance of *ODC* increased 2 h after differentiation was induced and decreased after that time (Figure 5). Taken together, these data demonstrate that treatment with polyamine precursors effects abundance of *ODC* but has no effect on *AMD1* temporally. 

### 3.5. The Effects of Polyamines on Abundance of mRNA Related to Satellite Cell Differentiation

**Figure 6:**
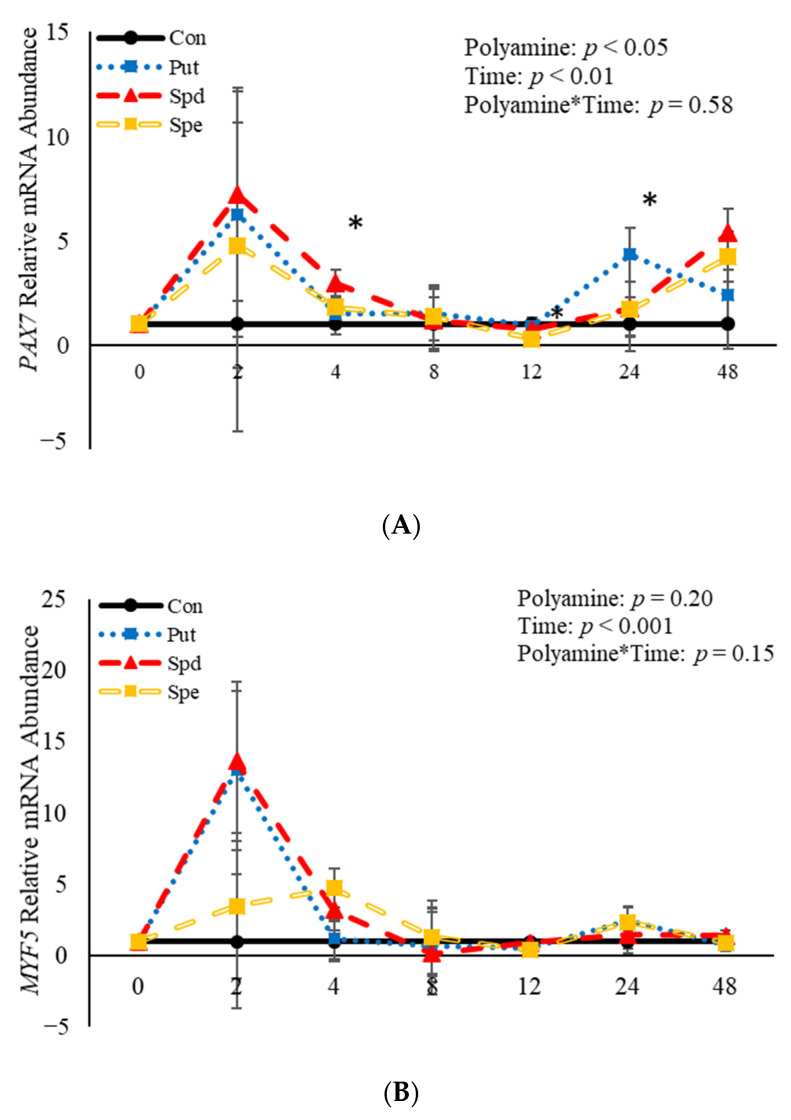

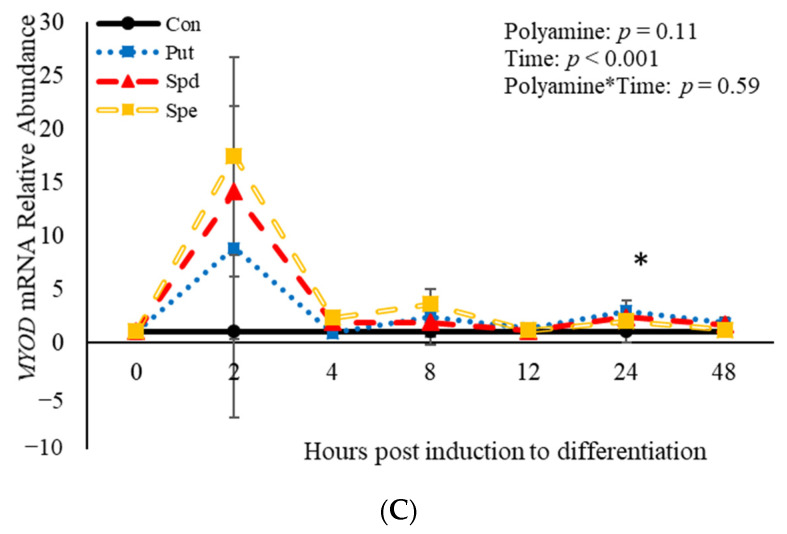
Relative mRNA abundance of paired box transcription factor 7 (*PAX7*; (**A**)*,* myogenic factor 5 *(MYF5;* (**B**), and myogenic differentiation factor 1 *(MYOD*; (**C**) from primary bovine satellite cells cultures following treatment with 2 mM putrescine (PUT), 1.5 mM spermidine (SPD) or 0.5 mM spermine (SPE). Cultures were grown to 80% confluency and treated with DMEM/3% horse serum and 2 mM PUT, 1.5 mM SPD, or 0.5 mM SPE. Abundance was measured 0, 2, 4, 8, 12, 24 and 48 h after treatment, as described in the Materials and Methods. The main effects of polyamine, time and polyamine*time in mRNA abundance were analyzed as repeated-measures. Data represent relative mRNA abundance normalized against 18S abundance and are presented as LS mean ± SEM from six separate replicates utilizing BSCs isolated BSCs isolated from at least three different animals. Differences (*p* < 0.05) between polyamines and the control cultures are indicated by a * at that particular time point.

No effects (*p* > 0.05) of the interaction between polyamine*time were observed relative to mRNA abundance of *PAX7*, *MYF5*, or *MYOD* (Figure 6). However, treatment with polyamines affected (*p* = 0.05) abundance of *PAX7* when analyzed as a repeated-measures (Figure 6A). Specifically, abundance of *PAX7* was increased (*p* = 0.02) 4 h post-induction to differentiate by SPD (Figure 6A), and 24 h (*p =* 0.002) post-induction to differentiate by PUT (Figure 6A). Despite the increase in abundance by SPD and PUT, SPE decreased (*p* = 0.04) abundance of *PAX7* 12 h post-induction to differentiate (Figure 6A) compared to control cultures. Although there was no effect (*p* > 0.05) of polyamine on abundance of *MYF5* when analyzed as a repeated measure, SPE increased (*p* = 0.02) abundance of *MYF5* 4 h post-induction to differentiate, but decreased (*p* = 0.05) abundance of *MYF5* 12 h post-induction to differentiate when compared to control-treated cultures (Figure 6B). Similarly, there was no effect (*p* > 0.05) of polyamine treatment on abundance of *MYOD* when analyzed as a repeated-measures (Figure 6C). However, PUT increased (*p* = 0.04) abundance of *MYOD* 24 h post-induction to differentiate (Figure 6C). Additionally, abundance of *PAX7, MYF5* and *MYOD* were each affected (*p* < 0.05) by time. Abundance of *PAX7* increased (*p* < 0.05) 2 h post-induction to differentiate then decreased after that before increasing (*p* < 0.05) again 48 h post-induction to differentiate (Figure 6A). Myogenin was not expressed in any of the cultures at any time point assessed. Abundance of both *MYF5* and *MYOD* mRNA increased 2 h post-induction to differentiate then decreased after that time (Figure 6B,C). Overall, these data demonstrate that polyamines generally increase expression of genes involved in BSC differentiation in a temporal manner. 

### 3.6. The Effects of Polyamines on Abundance of mRNA Involved in the Polyamine Biosynthesis Pathway

**Figure 7:**
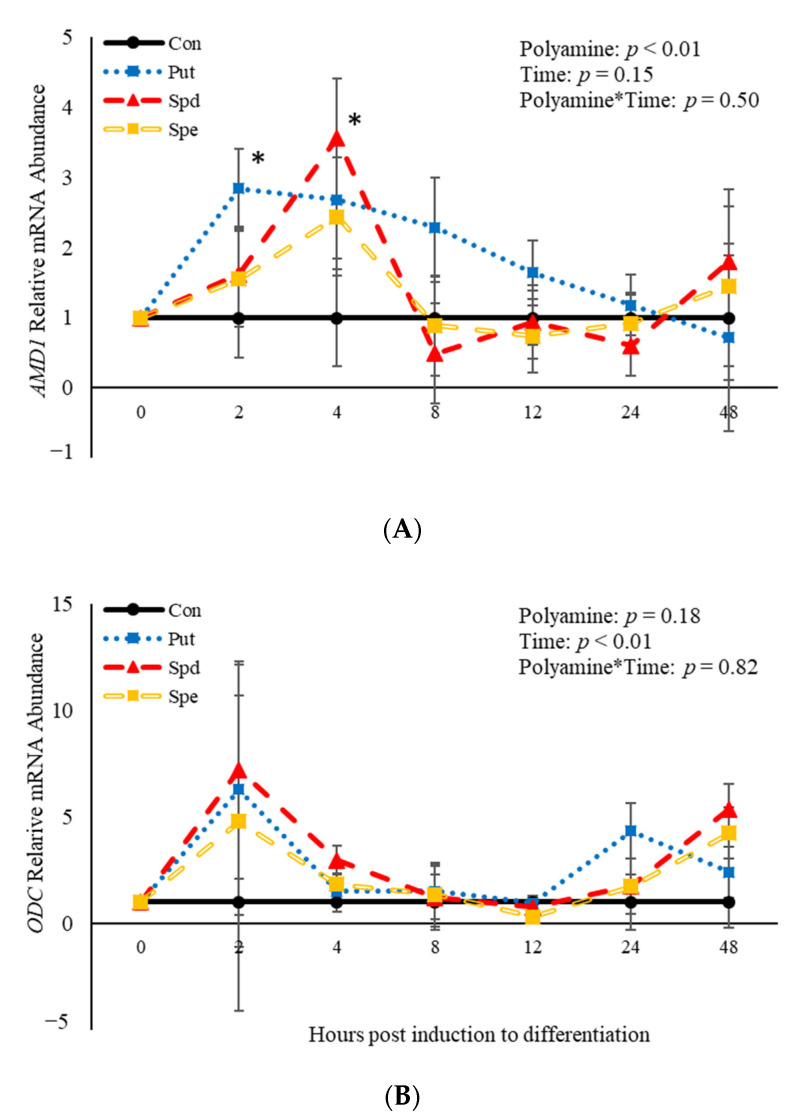
Relative mRNA abundance of *S-adenosylmethionine decarboxylase* (*AMD1;* **A**) and *ornithine decarboxylase (ODC;* **B**) from primary bovine satellite cells cultures following treatment with 2 mM putrescine (PUT), 1.5 mM spermidine (SPD) or 0.5 mM spermine (SPE). Cultures were grown to 80% confluency and treated with DMEM/3% horse serum and 2 mM PUT, 1.5 mM SPD, or 0.5 mM SPE. Cultures were grown to 80% confluency and treated with DMEM/3% horse serum and 10 nM TBA, 10 nM E2, or 10 nM E2/TBA. Abundance was measured 0, 2, 4, 8, 12, 24 and 48 h after treatment, as described in the Materials and Methods. The main effects of polyamine, time and polyamine*time in mRNA abundance were analyzed as repeated-measures. Data represent relative mRNA abundance normalized against 18S abundance and are presented as LS mean ± SEM from six separate replicates utilizing BSCs isolated BSCs isolated from at least three different animals. Differences (*p* < 0.05) between polyamines and the control cultures are indicated by a * at that particular time point.

No polyamine*time interactions (*p* > 0.05) were observed relative to mRNA abundance of *AMD1* or *ODC* (Figure 7). However, there was an effect (*p* = 0.04) of polyamine treatment where PUT increased (*p* = 0.02) abundance of *AMD1* 2 h post-induction to differentiate (Figure 7A). Additionally, SPD increased (*p* = 0.02) abundance of *AMD1* compared to control cultures 4 h post-treatment (Figure 7A). There was no effect (*p* > 0.05) of time on abundance of *AMD1* (Figure 7A). In contrast, there was an effect (*p* = 0.008) of time on abundance of *ODC*. However, there was no effect (*p* > 0.05) of polyamine treatment (Figure 7B). Abundance of *ODC* increases (*p* < 0.05) 2 h post-induction to differentiate then decreased after that time before increasing (*p* < 0.05) again at 48 h (Figure 7B). The data presented here demonstrate that *ODC* is not altered by polyamines, while both PUT and SPD alter mRNA abundance of *AMD1* in BSCs induced to differentiate.

## 4. Discussion

Understanding mechanisms of skeletal muscle growth is imperative to be able to increase both economic and environmental sustainability of the beef industry. Anabolic implants are used in the U.S. to improve growth of beef cattle, which increases the environmental and economic sustainability of the beef industry [3,7,12,41], but their mechanisms of operation are unknown. Testosterone and E2 have both been shown to influence the polyamine biosynthesis pathway [23,24,42,43], with emerging research suggesting that TBA may interact with the polyamine biosynthesis pathway to increase growth [25]. Polyamines are amino acids derivatives necessary for normal cellular proliferation and differentiation [27,32]. In cattle, the relationship between polyamines and growth remains ill-defined. The concentrations used to treat satellite cells in this study were determined based on previous research examining physiological polyamine concentrations in bovine lymphocytes [27]. Emerging research demonstrates that in BSCs, polyamines enhance proliferation rates [25], although their effect on differentiating BSCs is currently unknown. Therefore, the purpose of this research was to investigate the effects of polyamines, polyamine precursors, and steroidal hormones on mRNA abundance of BSCs induced to differentiate, as satellite cells must be able to differentiate in order to fuse into myotubes or with existing fibers properly [44]. Furthermore, we aimed to determine the effects that each of these molecules has on the polyamine biosynthetic pathway. 

In the present study, the effects of the steroidal hormones found in anabolic implants (E2 and/or TBA) on abundance of mRNA involved in the early stages of BSC differentiation (*PAX7, MYF5*, and *MYOD*) were investigated within the first 48 h of induction of differentiation. Investigating differentiation in the first 48 h of induction is important, as changes in myoblast differentiation have been reported to occur before 24 h [45]. A potential limitation of this study is that protein expression was not also investigated, as gene abundance can be influenced by the stability of the transcript and rate of transcription [46]. However, satellite cell activity may be evaluated using the mRNA abundance of myogenic regulatory factors [47], with measuring mRNA abundance to examine myogenic regulatory factors being routinely used by our lab group [25] and other lab groups [22,48] when studying satellite cells. Additionally, as this study is investigating the transition that occurs early on in BSCs when induced to differentiate, measuring mRNA abundance is the best option, as it is more sensitive to detect these changes and occurs before protein expression. The earliest changes that occur in differentiation are *Pax7* decreasing, while abundance of *Myf5* and *MyoD* increases [6,9,10]. The increase in *MyoD*, is then eventually followed by an increase in myogenin, but this happens much later in differentiating satellite cells [45]. In human skeletal muscle myoblasts, changes in myogenin are not detected until 72 h post-induction to differentiate [45], in rat satellite cells, myogenin peaks 3 d after being induced to differentiate [49], and in BSCs, myogenin is barely detectable after 48 h in differentiation media [50]. Due to the fact that myogenin is barely detectable after 48 h in BSCs induced to differentiate, when mRNA abundance of myogenin was measured in this study it was too early for it be expressed as this study was looking at the mRNA tanscripts in the first 48 h of BSCs induced to differentiate. In the current study, when the BSCs were induced to differentiate, *PAX7* mRNA abundance increased 2 h post-induction, and then proceeded to decrease in abundance. One possible explanation for this is that satellite cells are a heterogeneous population, with a portion of cells remaining in reserve and proliferating, not differentiating [8]. Additionally, the compounds used in this study have been found to increase proliferation rates of BSCs [25], and therefore it would not be surprising if some of the cells remained in a proliferative phase initially when being induced to differentiation. Another possible explanation for the variation observed in this study at 2 h is that even though the cultures used in this study were pure, they were primary cells, as previously mentioned satellite cells are a heterogeneous population of cells [5]. Therefore, it quite possible that even though the cells were induced to differentiate, the BSCs could have been in different stages of the myogenic process. The results of the present study demonstrate that treatment of BSC cultures induced to differentiate with E2 and/or TBA did not impact mRNA abundance of *PAX7*, *MYF5* or *MYOD* at the time points measured when analyzed as repeated-measures. However, when contrasts were performed on each treatment relative to the control, cultures treated with E2/TBA increased abundance of *MYOD* 8 h post-induction of differentiation. Furthermore, *MYOD* abundance was increased by E2. Estradiol also increased abundance of *PAX7,* while TBA did temporally enhance *MYF5* abundance when compared to control cultures. Other research has demonstrated that testosterone accelerates differentiation in C2C12 myoblastoma [51], as well as pushing C3H 10T1/2, pluripotent cells, towards a myogenic lineage [52]. Further, androgens may enhance cellular differentiation in C2C12 cells through the androgen-androgen receptor signaling pathway leading to an increase in expression of myogenin, without altering *MyoD* [33]. Additionally, when muscle derived stem cells are isolated from fetal calves at 180 d of gestation and treated with TBA, there is an increase in MYOD protein, which the authors postulated was mediated through the androgen receptor [53]. Previous research also found that BSCs treated with TBA or E2 had increased fusion indexes at 48 and 72 h post-differentiation when compared to control cultures [22]. However, *PAX7* and *MYOD* mRNA abundance was not altered in these differentiated BSC cultures. Additionally, in porcine satellite cells treated with testosterone, differentiation has been shown to be decreased [54]. These previous studies contrast with the results of the present study in that TBA, a testosterone analog, did not impact abundance of *MYOD, MYF5* or *PAX7* at the time points measured. However, the present study also found when BSCs are induced to differentiate and are treated with E2 and E2/TBA, there is an increase in mRNA abundance of these genes at specific time points suggesting that E2 may also play a role in differentiation of bovine satellite cells. This corresponds to previous research demonstrating that E2 and TBA increase fusion indexes of BSCs at 48 and 72 h post-differentiation [22]. However, in mice satellite cells, E2 has been shown to inhibit myogenesis by repressing the fusion of myoblasts into myotubes [55]. These findings suggest that steroidal hormones may impact abundance of mRNA involved in BSC differentiation at certain time points. One explanation for the lack of results presented in the present study is that it is not uncommon for delayed differentiation to occur in satellite cells, when proliferation and muscle mass are increased [54,56,57,58]. However, additional research is needed to further understand how treatment of BSCs with the steroidal hormones found in anabolic implants alters BSC differentiation.

The present study also assessed whether TBA, E2 or TBA/E2 affects mRNA abundance of *AMD1* or *ODC.* The present study found that TBA, E2 and TBA/E2 increase abundance of *AMD1* and *ODC* temporally in differentiating BSCs. Previous research has shown that a single injection of E2 increases *Odc* within 24 h in rats [23], while in proliferating breast cancer cells, E2 increased abundance of *Odc* and polyamine concentrations [24]. Mice that have been castrated have a decreased abundance of *Odc*, yet when these castrated mice are treated with testosterone, *Odc* abundance increases [59]. In previous research conducted utilizing proliferating BSCs, TBA does not alter abundance of *AMD1* or *ODC* [25]. Increased expression of ODC is thought to promote myoblast proliferation, but delay myogenic differentiation [60]. In proliferating BSCs isolated BSCs isolated from finished steers, ORN and MET have both been found to increase abundance of *ODC* [25]. In this research, *ODC* abundance was decreased by ORN 24 h post-induction to differentiate. This correlates with the increased abundance of *MyoD* 24 h post-treatment in ORN-treated cultures, suggesting that the decrease in *ODC* that has been observed in myogenic differentiation [60] occurs in differentiating BSCs.

Methionine and ORN were found to increase mRNA abundance of *PAX7*, *MYF5*, and *MYOD* temporally in differentiating BSCs. In vitro studies examining MET and ORN in mammalian cells are limited, with nearly none occurring in livestock species. The concentration of MET used in this study was chosen based off previous research conducted by our lab group determining the optimal concentration to increase BSC proliferation [25]. This is important in an ovine model, as growth of cultured satellite cells is extremely sensitive to the amount of MET in the medium [61]. A number of research trials have used in vivo studies to investigate MET supplementation. When MET is supplemented to Holstein feeder steers, it has been found that gain efficiency increases linearly with MET supplementation [62]. The varying degrees of MET supplementation impacting differentiation suggests that an optimal MET concentration needs to be determined at different stages of growth to optimize growth of beef. Methionine is known to be a limiting amino acid in beef [63]. Therefore, when looking at whole animal studies, it is difficult to determine whether changes in growth are attributed to satellite cell differentiation, protein synthesis, or effects on other systems within the body. Ornithine has been shown to assist with increasing protein synthesis in skeletal muscle of humans post-surgery [64]. Furthermore, MET and ORN can be utilized as an energy source when metabolized [65,66], which may alter energy balance within the cell resulting in altered differentiation. As such, more research needs to be performed to determine the mechanism through which MET and ORN may be altering differentiation of primary BSCs.

When polyamine precursors are decarboxylated, polyamines are produced [28]. Polyamines are orally active and can be derived from the diet, or endogenous production in the tissues through the polyamine biosynthesis pathway [26,28]. This research found that SPD increased *PAX7* mRNA abundance 4 h post-treatment compared to controls. Spermine did decrease *PAX7* abundance 12 h post-treatment in the present study. Additionally, we found that 4 h post-treatment, TBA and SPE both increased *MYF5* abundance, an important regulator of satellite cell differentiation [44].

Additionally, the present study examined the impact of polyamines on mRNA abundance of the rate limiting steps in the polyamine biosynthesis pathway. When differentiating BSCs are treated with the polyamines, PUT and SPD, abundance of *AMD1* is increased. However, treatment with polyamines had no effect on *ODC* abundance. Other research has found that when proliferating BSCs are treated with SPD or SPE, *AMD1* abundance is increased [25], while abundance of *ODC* is not altered [25]. Additional research needs to be completed to determine how the polyamines impact both growth of skeletal muscle and the polyamine biosynthetic pathway in beef animals.

The overall finding of this research suggests that steroidal hormones largely do not impact mRNA abundance in BSCs induced to differentiate, but polyamines and their precursors do increase abundance of genes related to BSC differentiation. Furthermore, steroidal hormones, polyamine, and polyamine precursors were each found to impact the polyamine biosynthetic pathway. Therefore, future work needs to investigate the interplay of these growth promotants and the polyamine biosynthesis pathway on cattle skeletal muscle as a whole, as it is difficult to ascertain whether increased growth comes from increased proliferation, differentiation, protein synthesis, other mechanisms or a combination of these factors.

## Figures and Tables

**Table 1:** Primer and Probe Sequences used in Real-Time PCR.

Abbreviation	GBA ^1^ Number	Primers and Probe Sequences, 5′-3′
*18S* ^2^	AF243428	FP: CCACGCGAGATTGAGCAAT
		RP: GCAGCCCCGGACATCTAA
		TP: ACAGGTCTGTGATGCC
*PAX7* ^3^	XM_616352.4	FP: AGGACGGCGAGAAGAAAGC
		RP: CCCTTTGTCGCCCAGGAT
		TP: AAGCACAGCATCGAC
*MYF5* ^4^	NM_174116.1	FP: AGCCCCACCTCAAGTTGCT
		RP: GCTGTCAAAACTGCTGCTCTTTC
		TP: CATGCCTGAATGTAAC
*MYOD* ^5^	NM_001040478.2	FP: CCGCCTGAGCAAAGTCAAC
		RP: GGGCAGCCGCTGGTTT
		TP: TGCACGTCTAGCAACC
*MYOG* ^6^	XM_001790098.1	FP: CCGCCACGCTGAGAGAGA
		RP: GGCCTCGAAGGCTTCATTC
		TP: GGCCTCGAAGGCTTCATTC
*ODC* ^7^	NM_174130	FP: CTGTACTGATCCTGAGACCTTTG
		RP: GCTTTACATCCTCTGATCCAGG
		TP: ATCTCTGATGCCCGCTGTGTCTTT
*AMD1* ^8^	NM_173990	FP: TGCTGGAGGTTTGGTTCTC
		RP: TCAAAAGTATGTCCCACTCGG
		TP: TTGGTTTGCGTCGGGTTGCTG

Forward primer (FP), reverse primer (RP) and TaqMan^®^ probe (TP) sequence along with gene bank accession (GBA) number for the genes analyzed by employing TaqMan^®^ primer probe system of real-time PCR. ^1^ GenBack Accession Number ^2^ Ribosomal 18S. ^3^ Paired box transcription factor 7. ^4^ Myogenic factor 5. ^5^ Myogenic differentiation factor 1. ^6^ Myogenin. ^7^ Ornithine decarboxylase. ^8^ S-adenosylmethionine decarboxylase.

## Data Availability

The data presented in this study are available upon request from the corresponding author. The data are not publicly available due to restrictions in place by the funding agency.
